# Minimally invasive interventions for intracranial pathologies using tubular retractors in the pediatric population: Safety, efficacy, technical aspects and outcomes

**DOI:** 10.1371/journal.pone.0315744

**Published:** 2025-03-10

**Authors:** Marian Michael Bercu, Andres F. Restrepo-Orozco, Leonard H. Verhey, Casey J. Madura, Anthony M. Avellino, Joseph A. Petronio, Paul A. Mazaris, Justin A. Singer

**Affiliations:** 1 Division of Pediatric Neurosurgery, Department of Clinical Neurosciences, Helen DeVos Children’s Hospital, Corewell Health, Grand Rapids, Michigan, United States of America; 2 College of Human Medicine, Michigan State University, Grand Rapids, Michigan, United States of America; 3 Division of Neurosurgery, Department of Clinical Neurosciences, Corewell Health, Grand Rapids, Michigan, United States of America; 4 The University of Arizona College of Medicine, Department of Neurosurgery, Tucson, Arizona, United States of America; 5 Department of Neurosurgery, Banner University Medical Center, Tucson, Arizona, United States of America; University of Marburg: Philipps-Universitat Marburg, GERMANY

## Abstract

**Background:**

Minimally invasive surgeries for intracranial pathologies are gaining popularity, recognizing the intrinsic benefits, mostly related to recovery time, while minimizing injury to healthy parenchyma and adjacent functional areas, especially during the resection of deep and centrally located lesions. These procedures require technical familiarity and cultivated surgical experience, coupled with dedicated instruments, appropriate planning, and a stringent patient selection.

**Objective:**

To describe our novel experience with minimally invasive trans-sulcal parafascicular surgery (MIPS) in a single-center pediatric population, emphasizing the interdependencies between surgical experience, best practices, preparation, and positive surgical outcomes.

**Methods:**

This single center retrospective review included an electronic medical record (EMR) retrieval of all pediatric patients undergoing minimally invasive trans-sulcal parafascicular surgeries (MIPS) between 2018 and 2023. Clinical, demographic, and radiographic data were captured as were previous surgical procedures, operative approach and technique, operative duration, post-operative day discharge (POD) and length of follow up. Outcomes, including complications and the need for additional interventions, are reported.

**Results:**

A total of 27 consecutive procedures, treating 22 patients aged 10-months to 19-years were evaluated. Treated pathologies included tumors, vascular lesions, infections, hemorrhage, and hydrocephalus, with the average follow-up > 19 months. Surgical outcomes were similar, if not superior to, the standard of care, considering the extent of resection of various types of lesions, evacuation of hematoma or abscess, as well as complex fenestrations. MIPS procedures were successfully used in a subgroup of patients previously undergoing operations with “standard” approaches. No patients experienced direct complications as a result of the procedure. Recovery times were shorter and the procedure itself was better tolerated in comparison to classical interventions.

**Conclusions:**

This largest reported pediatric series using MIPS for a variety of pathologies, demonstrates the feasibility, safety, and possibly superior outcomes in children. Technical familiarity and development of surgical experience with MIPS is critical to optimal outcomes.

## Introduction

The use of minimally invasive techniques for the surgical treatment of adult neurosurgical conditions, such as intracranial hemorrhage (ICH) [1], ventricular or deep-seated tumors [2] and vascular malformations [3] continues to gain popularity. Case reports, as well as larger case series [4], demonstrate that in experienced hands, very complex and deep-seated pathologies can be safely approached, debulked or fully resected, with similar, if not lower, complication rates, coupled with shorter and better tolerated recovery. The inherent advantages of tubular retractors, that include minimizing collateral damage to normal brain and vasculature [5], and maintenance of a stable working corridor, occasionally larger than the one available using classic neurosurgical approaches (such as interhemispheric, supra cerebellar-infratentorial, etc.), should be counter-balanced by the increased complexity of those approaches, the required learning curve, need for dedicated instruments, and the adoption of a new conceptual approach.

The pediatric developing brain is occasionally considered to be more resilient to injury than the adult brain, most likely secondary to the ability for re-modulation and gain-of-function [6–8]. Nonetheless, the young brain tissue is typically more friable and therefore, more susceptible to collateral injury. Larger craniotomies can be associated with significant bony defects (with associated growth deformities), and, especially in younger patients, classical approaches pose elevated difficulty and risks associated with positioning such as the limited ability to use rigid fixation frames, smaller working corridors, strict limitations on blood loss and other unique factors [9]. Surgical methods that can minimize procedure length and favor shorter post-surgical recovery, in terms of hospitalization interval, as well as reduced need for prolonged rehabilitation are beneficial in the pediatric population, reducing long-term developmental delays, psychological effects such as anxiety, and improving the overall experience.

Minimally invasive parafascicular surgery (MIPS) for pediatric lesions has gained popularity in our institution during the last five years, after recognizing the significant benefits and excellent outcomes, in parallel to the very limited complication profile. However, the benefits and best practices, including technical aspects of using MIPS in the pediatric population with varied pathologies, have not been thoroughly characterized.

Herein we report the largest pediatric series of MIPS procedures in 22 patients, between 10-month-old to 19-years-old, with a variety of pathologies, including benign conditions, vascular malformations, tumors, colloid cysts, and infections. The feasibility, safety, benefits and post-surgical outcomes associated with MIPS in a pediatric population are described; interdependencies between technical familiarity and surgical experience with MIPS, along with appropriate patient selection, are emphasized.

## Methods

This single center retrospective review included all pediatric patients undergoing MIPS between 2018 and 2023 at Helen DeVos Children’s Hospital. Institutional review board (IRB) approval was obtained for the retrospective chart review, further collecting the EMR data between 30/01/2022 and 15/03/2024. The IRB waived the requirement for informed consent.

Surgical pre-planning included identifying the most favorable patient position to reach the lesion via the identified surgical trajectory. Trajectories were planned using the StealthStation™ S8 neuronavigation system (Medtronic, Minneapolis, MN), with the further goal to minimize disruption to white matter tracts. All surgeries were performed by neurosurgeons experienced with tubular retraction systems.

All procedures implemented a transsulcal approach using the BrainPath^®^ tubular retractor system (NICO Corporation, Indianapolis, IN), with obturators and sheaths measuring from 11x50 mm to 13.5x75 mm (occasionally more than one size during the same procedure), with or without the Myriad^®^ microdebrider system (NICO Corporation, Indianapolis, IN). BrainPath® retractor length was determined based on the distance from the calvarial surface to the lesion. For most patients, a 13.5x60 mm obturator and sheath were selected. The smaller sheath (11 mm in diameter) was used for specific pathologies, such as complex fenestrations in very young patients, evacuation of abscess or hematoma or for less complex intraventricular pathologies. The tubular sheath required intraoperative replacement from a 13.5x50 mm sheath to a 13.5x60 mm sheath in two cases, both during resection of intraventricular tumors, given the collapse of the CSF spaces and, as the resection progressed and deepened.

The procedures were completed under high magnification, using standard microscopy (Zeiss™) or a 3-dimensional exoscope (Aesculap, AEOS^®^). Rigid or flexible endoscopes were used through the sheath in several cases to determine residual lesion, safety of additional resection, including for a better definition of the intraventricular anatomy.

Neuromonitoring was extensively used, with similar indications to standard of care. Optical or electromagnetic (EM) neuronavigation (StealthStation™ S8) was used in all cases. For the more recent cases requiring EM neuronavigation, the BrainPath Navigation Probe Adapter^®^ (NICO Corporation, Indianapolis, IN) was used. Advanced imaging, including functional MRI (fMRI)-based tractography and magnetoencephalography (MEG)-defined functional areas, were implemented in preoperative planning, identifying safe corridors towards the deep located lesions, as well as intraoperatively during the resection through the tubular retractor. Pre-operative 5-Aminolevulinic acid (5-ALA, Gleolan^®^) was administered in two cases.

Clinical, demographic, and radiographic data were obtained including age, sex, presenting neurologic deficits, MRI findings including lesion location and size, and previous surgical procedures. Intraoperatively, the following were captured: operative approach or trajectory, BrainPath® endoport length (mm), operative duration (minutes), use of intraoperative MRI; additionally post-operative day discharge (POD) and length of follow up (days) are reported (Table 1).

**Table 1:** MIPS experience: Demographic data, procedures, and outcomes.

Pt.	Age	S	Pathology	Presentation	MRI findings	Previous surgical procedures	Procedure	BrainPath size(mm)	Post-op imaging	Post-op complications or additional interventions	Outcome	Length of procedure (min)	Intra-op MRI	D/C POD	Length of folow-up
1	11	M	Subependymoma WHO I	Work-up for TSC d/t skin lesions; Dx.: NF2	T2 and post gadolinium hyperintense left posterior temporal stem lesion with restricted diffusion; 2.2 x 1.8 x 1.8 cm. Slowly growing on f/u images. The lesion is splaying the arcuate fasciculus and inferior longitudinal fasciculus.	None	Left parieto-occipital approach with EVD placement	13.5x75	GTR	None	Slow-growing limited recurrence; conservative management.	157	Y	2 (EVD)	1251
2	10	F	Anaplastic Ependymoma WHO III	PHx. of posterior fossa Ependymoma	Mildly lobulated enhancing nodule within the anterior third ventricle at the level of the foramina of Monro, slightly crossing into the frontal horn of the left lateral ventricle, measuring approximately 9 mm AP by 7 mm transverse by 11 mm craniocaudal	S/p Resection of posterior fossa ependymoma X2; S/p ablation of posterior fossa recurrent ependymoma	Left frontal transventricular approach for resection of 3^rd^ ventricular mass	11x60	GTR	None	Posterior fossa re-do craniotomy for tumor recurrence (X2). New metastatic pineal area lesion. Spinal metastases; post resection. No anterior 3^rd^ ventricular residual/recurrence or hydrocephalus	67	N	2	677
3	17	M	Cavernoma	Seizure	Right parietal deep-seated cavernoma, bordering the lateral ventricle (subependymal)	None	Right parietal transcortical approach	13.5x60	GTR	None	Seizure-free, no neurological deficits	131	N	2	371
4	17	F	Ependymoma WHO II	TBI, post-concussion, incidental finding	Nonehnhancing, T2 hyperintense lobular mass in the body of the left lateral ventricle, with anterior component nearing the foramen of Monro, measuring 3.3 x 1.5 x 1.5 cm and mildly expanding the portion of the left ventricle where it is located; mild mass effect on the adjacent septum pellucidum and forniceal pillars, that were slightly displaced rightward	None	Right frontal approach with septostomy for resection of left ventricular tumor	13.5x75	GTR	Flat-affect, short term memory recall deficits (post-TBI), temporary leg fasciculations - resolved	No neurological deficits, no evidence of residual/recurrence at last f/u	164	Y	3	660
5	10	M	Colloid cyst	Exertion-induced syncopal events for 1 month prior to diagnosis	A 10 x 10 x 8 mm non-enhancing T1 and T2 hyperintense lesion near the foramen of Monro, cradled by the anterior columns of the fornix and septum pellucidum (3^rd^ ventricular lesion)	S/p Aborted endoscopic resection attempt d/t unfavorable anatomy	Left frontal transcortical transventricular approach for resection of a 3^rd^ ventricular colloid cyst	13.5x60	GTR	None	No new neurological deficits after the procedure, no evidence of residual/recurrence at last f/u	128	N	1	1905
6	6	M	Tanycytic Ependymoma WHO II	Increased ICP symptoms, papilledema	A 3.6 x 2.3 x 3.7 cm mass centered in the posterior third ventricle extending into and filling the aqueduct of Sylvius. Lesion demonstrates FLAIR hyperintensity, mildly decreased diffusion, no enhancement. The tectum appears thinned and displaced posteriorly. Marked obstructive hydrocephalus	Pre-operative placement of EVD	Right frontal transcortical transventricular approach; use of flexible endoscope for intermittent evaluation of lesion	13.5x60	STR - approximately 80% resection	CSF leak from incision post-EVD wean, oversewn	Lesion stable during the f/u, resolved papilledema and no CSF outflow obstruction. No neurological deficits	260	N	14 (EVD)	1412
7	8	M	Cavernoma	Intermittent headaches, concurrent viral infection	Multiseptated non-enhancing T1 hyperintense medial left parietal lesion with blooming artifact measuring 4.1 x 3.0 x 2.5 cm. Moderate adjacent vasogenic edema in the posterior cerebrum but also involving the splenium of the corpus callosum. fMRI: Fibers of the left inferior frontal occipital fasciculus tract are passing just lateral and inferior to the parietal mass	None	Prone, Left parieto-occipital approach	13.5x60	GTR	None	No neurological deficits, no evidence of residual/recurrence at last f/u	175	Y	3	1257
8	4	F	Cavernoma	Vomiting, lethargy, decreased oral intake	Hemorrhagic, multicystic lesion in the deep left cerebral hemisphere deforming the globus pallidus and thalamus, with a thin rim of T2 hyperintense signal (measuring approximately 1-1.5 mm) separating it from the third ventricular lumen. The lesion measures 33 x 26 x 29 mm; consists of scattered hemorrhagic locules, all demonstrating T2 hypointensity consistent with acute/subacute hemorrhage. No abnormal enhancement	None	Right frontal transcortical transventricular approach for resection of left thalamic suspected cavernoma	13.5x60	Presumed GTR (intra-op and post-op MRI)^*^	See below- additional MIPS procedure. Superficial skin fibrosis w/ debridement (no infection)	Recurrent hemorrhage from unidentified residual cavernous malformation	197	Y	9 (EVD)	1307
8	5	F	Cavernoma	Routine f/u; several days of unexplained irritability, headaches, decreased activity	Minimally septated predominantly non-enhancingT1 hyperintense with mixed T2 signal and susceptibility lesion, abutting the left thalamus, measuring 3.2 x 2.7 x 2.3 cm. Small halo of vasogenic edema	See above	Right frontal transcortical transventricular approach for resection of left thalamic cavernoma	13.5x60	Presumed GTR (intra-op MRI difficult to interpret)	See below- additional MIPS procedure	Post-op MRI: Residual left thalamic hematoma and/or cavernoma with interval decrease in size compared to pre-op MRI (2.4 x 2.0 x 2.2 cm)	187	Y	8 (EVD)	
8	5	F	Cavernoma	Post-op full MRI	Residual left thalamic hematoma and/or cavernoma with interval decrease in size compared to pre-op MRI (2.4 x 2.0 x 2.2 cm)	See above	Right frontal transcortical transventricular approach for resection of left thalamic cavernoma	13.5x60	GTR	No additional interventions	No residual/recurrent cavernoma, no neurological deficits	173	Y	3 (EVD)	
9	15	F	Cavernoma	Fall with TBI	Granular T1 and T2 hyper- and hypointense lesion involving the right genu valgum and bilateral fornices measuring 2.6 x 1.6 x 2.1 cm. Developmental venous anomaly along the superior aspect of lesion drains into the internal cerebral vein	None	Awake right frontal transcortical transventricular approach	13.5x60	GTR	None	Normal neuropsychological evaluation with no neurological deficits. Academic excellence	243	Y	5 (EVD)	968
10	7	M	Atypical Meningioma WHO II	Evening headaches and emesis, papilledema	Solid and cystic neoplasm with central calcifications and epicenter in the deep left hemisphere, measuring 5.6 x 3.2 x 5.3 cm. Solid component is isointense to gray matter on T1 and mildly hypointense and T2 with moderate enhancement. Mass effect on the atrium of the left lateral ventricle, posterior third ventricle and quadrigeminal plate cistern	Pre-operative placement of EVD	Left posterior parietal transcortical approach for resection of intra-ventricular calcified mass	13.5x50, 13.5x60	GTR	VPS (1 month after resection)	No neurological deficits, no evidence of residual/recurrence at last f/u	575	Y	10 (EVD)	1105
11	5	M	Pilocytic Astrocytoma, WHO I	Planned second stage intervention	Solid and cystic chiasmatic/hypothalamic neoplasm splays the circle of Willis and invaginates into the third ventricle measuring 5.6 x 5.1 x 3.9 cm. Dense flocculent enhancement of the solid component. Tumoral cysts, predominantly along the right lateral aspect of the lesion with the largest measuring 1.8 x 0.8 x 2.0 cm. Severe obstructive hydrocephalus	S/p Left extended pterional craniotomy for STR of pilocytic astrocytoma	Left frontal transcortical approach	13.5x60	Resection of additional 50% of tumor (approx. 80% total resection)	Chemotherapy	No new neurological deficits (previous left hemiparesis, stable); Stable residual mass at last f/u, no hydrocephalus	321	Y	20 (EVD)	972
12	4	F	Pilocytic Astrocytoma, WHO I	Progressive left-sided weakness, 4-/5	Right basal ganglia T2 hyperintense, T1 hypointense uniformly enhancing mass with cystic regions medially. The lesion measures 27 x 32 x 26 mm and extends into the temporal area; the corticospinal tract deflected antero-laterally per fMRI	None	Right temporal transcortical transventricular approach with 5-ALA (positive) and neuro-monitoring. Resection stopped d/t motor responses at 2 mA	13.5x60	≥70% resection of enhancing mass, stable	None	Improved weakness from presentation, minimal spastic gait, excellent function of upper extremity. Stable residual enhancing neoplasm in the deep margin of the resection	226	Y	4	712
13	11	F	Abscess, Strep. intermedius	Sinusitis, extension from oral cavity (dental source); right sided-dysmetria at presentation	Complex restricted diffusion collection adjacent to or within the superior medial right cerebellar hemisphere, measuring 3.1 x 3.5 x 2 cm. A more irregular component is extending superiorly dorsal and lateral to the mid brain which measures 1.3 x 1 x 2.2 cm	S/p Antrostomy, Frontal Sinusotomy, Ethmoidectomy and Sphenoidotomy	Suboccipital craniotomy for evacuation of cerebellar abscess	11x50	Full evacuation of cerebellar abscess	Re-formation of abscess, re-evacuation using EVD	Resolved dysmetria, possible new academic difficulties	83	N	9	392
14	1.5	M	Pilocytic Astrocytoma, WHO I	Emesis with FTT, bilateral optic nerve atrophy	Chiasmatic/suprasellar mass which measures 5.5 x 4.4 x 4.1 cm. The mass is T1 hypointense, T2 hyperintense and demonstrates significant post contrast-enhancement. The mass abuts the suprasellar internal carotid arteries and proximal anterior cerebral arteries without apparent narrowing. There is moderate to severe enlargement of the lateral ventricles with associated transependymal edema	None	Right frontal craniotomy with transcortical approach	13.5x50	Approximately 40% resection	Chemotherapy; Obstructive hydrocephalus requiring shunting, 15 months post-op; Additional resection of approximately 40-50% of recurrent mass through same MIPS tract. Laser ablation. Targeted chemotherapy	Significant developmental progress, resolved FTT; very minimal tumor enlargement vs. post-resection changes; completed chemotherapy with no change in tumor size, reduced enhancement	245	Y	9 (EVD)	677
15	0.83	F	Choroid plexus hypertrophy; Coffin-Siris syndrome; Dandy Walker variant	Intractable asciites with functioning VPS	Moderate to marked ventriculomegaly, particularly the frontal and temporal horns with operating shunt. Abdominal U/S: Fluid in the right upper quadrant measures 10.5 x 3.9 x 7.9 cm. Fluid in the right lower quadrant measures 8.5 x 8.7 x 6.8 cm. Fluid in the left upper quadrant measures 6.7 x 3.5 x 5.2 cm. Fluid in the left lower quadrant measures 7.1 x 4.8 x 5.5 cm	ETV, 4 shunt procedures	Left parietal craniotomy for resection of left lateral ventricle choroid plexus	11x60	Approximately 90% resection of left lateral ventricle choroid plexus	Limited subdural fluid collection, conservative management (increasing shunt valve resistance); resolved	Decreasing ventriculomegaly	132	N	14 (EVD)	375
16	8	M	Subependimal Giant Cell Astrocytoma	Blurry vision, papilledema	Intraventricular mass, within the right lateral ventricle superior and just anterior of the foramen of Monro. This mass avidly enhances and appears hypervascular with several internal curvilinear structures of particular high enhancement. Size: 25 x 24 x 27 mm (AP, TV, SI). Mass effect on adjacent structures including the right caudate nucleus, forniceal pillars and septum	None	Right frontal MIPS	13.5x50	GTR	Resolved small subgaleal collection	Slightly larger right ventricle, asymptomatic	490	Y	13 (EVD)	245
17	17	M	Trauma, ICH	MVA, GCS 3	Blossoming right frontal capsular contusion with significant mass effect, including midline shift	None	Right frontal mini craniotomy for ICH evacuation	11x70	Evacuation of 80-90% of ICH	Intra-op EVD	ICPs maintained within normal range (15-20) on maximal medical management; At last f/u patient has returned to cognitive baseline, ambulating with lower extremity brace only, distal upper extremity spastic paresis	119	N	37	396
18	2	F	IVH, Prematurity, Shunted hydrocephalus, Loculated hydrocephalus	Shunt malfunction, Multi-compartmental Post-hemorrhagic Hydrocephalus	Multi-septated hydrocephalus, multiple cystic loculations	>10 CSF-related procedures; Shunt with 3 intracranial catheters	Left posterior craniotomy with trans-sulcal approach for left-sided multiple fenestrations	11x60	Successful fenestration (CT Ventriculogram)	See below	Communicating hydrocephalus	145	N	30 (EVD)	363
18		F	IVH, Prematurity, Shunted hydrocephalus, Loculated hydrocephalus	Planned 2^nd^ stage	Multi-septated hydrocephalus, multiple cystic loculations	See above. Planned 2^nd^ stage	Right frontal craniotomy with trans-sulcal approach for right-sided multiple fenestrations and septostomy	11x75	Successful fenestration (CT Ventriculogram)	Shunt revision, single intracranial catheter	Communicating hydrocephlaus, shunt with single intracranial catheter; Proximal shunt revision after 2 months	154	N	23 (EVD)	
18		F	IVH, Prematurity, Shunted hydrocephalus, Loculated hydrocephalus	Routine f/u	Routine f/u, progressive bilateral multi-loculated (cystic), mostly temporo-parietal hydrocephalus	See above	Left temporal mini craniotomy with trans-sulcal approach for fenestration of multiple intracranial fenestrations	11x60	Successful fenestration	See below	See below	122	N	4	
18		F	IVH, Prematurity, Shunted hydrocephalus, Loculated hydrocephalus	Planned 2^nd^ stage	Routine f/u, progressive bilateral multi-loculated (cystic), mostly temporo-parietal hydrocephalus	See above. Planned 2^nd^ stage	Right temporal mini craniotomy, with microsurgical port-based fenestration of multiple intracranial cysts	11x60	Successful fenestration	Bilateral (temporo-parietal) cystic progressive dilation with mass effect (asymptomatic). Addition of bilateral parieto- temporal ventricular catheters (three-limb shunt) with excellent decompression, suggestive of patent fenestrations. Significant developmental progress at last f/u	Significantly decompressed temporo-parietal loculated hydrocephalus	150	N	2	
19	14	M	Diffuse midline glioma, H3K27-altered (H3K27me3 loss); p53 with diffuse expression, WHO IV	Obstructive hydrocephalus with intratumoral hemorrhage	Large hemorrhagic midline tumor, most likely originating from the thalamus/hypothalamus, with extension in both lateral and third ventricles; Obstructive hydrocephalus	Pre-operative placement of EVD	Right frontal transcortical approach for debulking and resection of acutely hemorrhagic tumor with acute herniation and clinical deterioration	13.5x60	Resection of approximately 40% of tumor (>50% of enhancing mass)	Right frontal transcortical craniotomy for NTR (>90%) of deep midline tumor. VPS	Additional open procedure for resection of > 95% of tumor. Tumor recurrence winthin 2 weeks, extensive re-growth in 6 weeks. Palliative care. Deceased 2.3 months post-op	218	N	44	71
20	8	M	Diffuse midline glioma, H3 K27-altered, p53 mutation, H3K37me3 loss	Hemiparesis, nausea and vomiting; Obstructive hydrocephalus with intratumoral hemorrhage, transfalcine herniation and hemiparesis	Contrast enhancing right thalamic tumor, infiltrative into the basal ganglia, midbrain and medulla oblongata. The mass results in midline shift of approximately 1.3 cm	None	Right parietal transcortical approach for debulking and resection of tumor with 5-ALA	13.5x50	Resection of 70-80% of enhancing thalamic mass	Radiotherapy; ETV followed by shunt at a different institution over 4 months after the initial sugery (after presenting with new-onset seizures)	Resection of 70-80% of enhancing thalamic mass; post-resection resolution of obstructive hydrocephalus. Progressive involvement of the brainstem. Deceased 5.5 months post-op	159	N	15 (EVD)	165
21	19	F	Pilocytic Astrocytoma, WHO Grade 1	Routine f/u, s/p resection of right temporal glioma, 2009	Left frontal subependymal T2 hyperintense nodule with restricted diffusion measuring 3.2 x 2.0 x 2.3 cm	2009: Resection of right temporal glioma and chemotherapy; 2019: biopsy of left basal ganglia low grade glioma	Left frontal trans-sulcal approach for resection of left frontal tumor with intraventricular extension	13.5x60	GTR	None	Subjective minimal post-operative temporary short-term memory difficulties; fully resolved, back to college	487	Y	3	53
22	16	F	Central Neurocytoma, WHO Grade 2	Papilledema and headaches	Intraventricular mass measuring approximately 2.0 x 2.0 x 2.8 cm within the anterior body of the left lateral ventricle, extending into the left foramen of Monro; predominantly isointense to slightly hyperintense on T2 weighted images, hyperintense on FLAIR images, and near isointense to brain parenchyma on T1-weighted images. Mild heterogeneous enhancement on post-contrast images	None	Left frontal trans-sulcal approach for resection of intraventricular tumor	13.5x50, 13.5x60	GTR	None	Subjectively reported short term memory difficulties when tired, continues to excell at school, including at college classes. Intermittent headaches without hydrocephalus	247	Y	17 (EVD)	92

Abbreviations: Pt. – patient number; S – sex; D/C – discharge; POD - post-operative day; TSC - Tuberous Sclerosis Complex; NF2 - Neurofibromatosis Type 2; Dx. – diagnosis; F/u – follow-up; EVD – external ventricular drain; N/A – not available; GTR - gross total resection; Y – yes; PHx. – patient history; TBI - Traumatic brain injury; AP – anteroposterior; S/p – status post; d/t – due to; N – no; STR – sub-total resection; F/u – follow-up; VPS – ventriculo-peritoneal shunt; 5-ALA – 5-aminolevulenic acid; FTT – failure to thrive; ETV – endoscopic third ventriculostomy; MVA – motor vehicle accident; GCS – Glasgow coma scale; ICH – intracranial hemorrhage.

* Urgent procedure (night-time).

** MRI completed intra-op with return to OR for additional resection.

The extent of postsurgical resection or additional outcomes were estimated based on the immediate post-operative images (MRIs); complications, the need for additional interventions and clinical outcomes are reported. The outcomes were evaluated based on the most recent available clinical follow-up, in all cases accompanied by updated imaging. Descriptive statistics were captured and evaluated.

## Results

**Fig 1:**
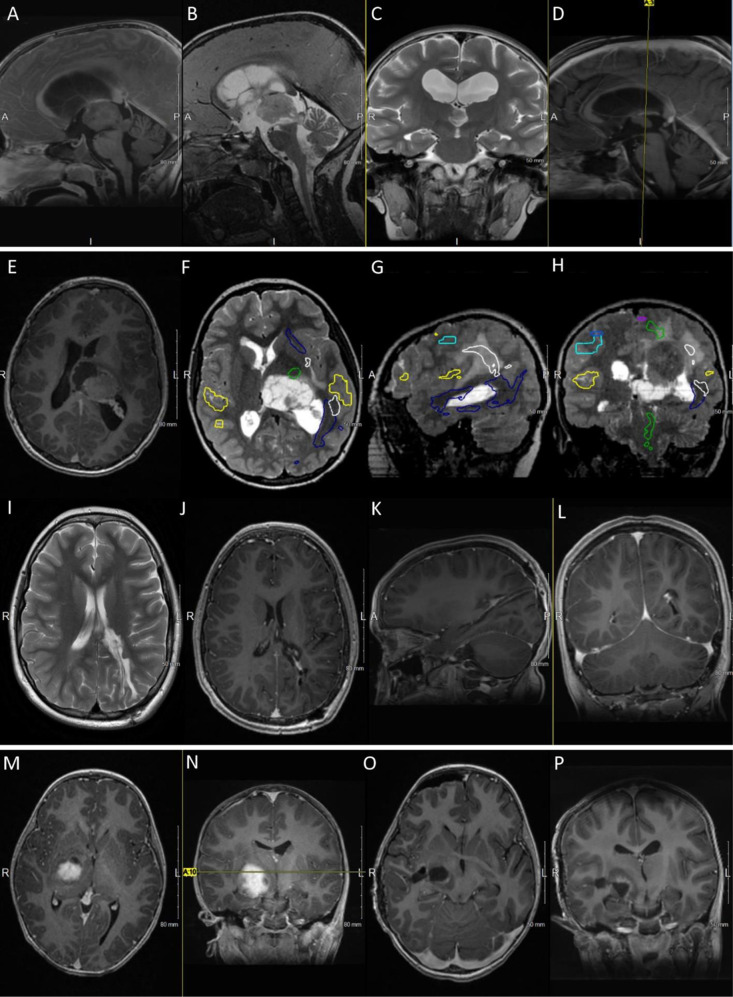
Illustrative cases of tumors.

**Fig 2:**
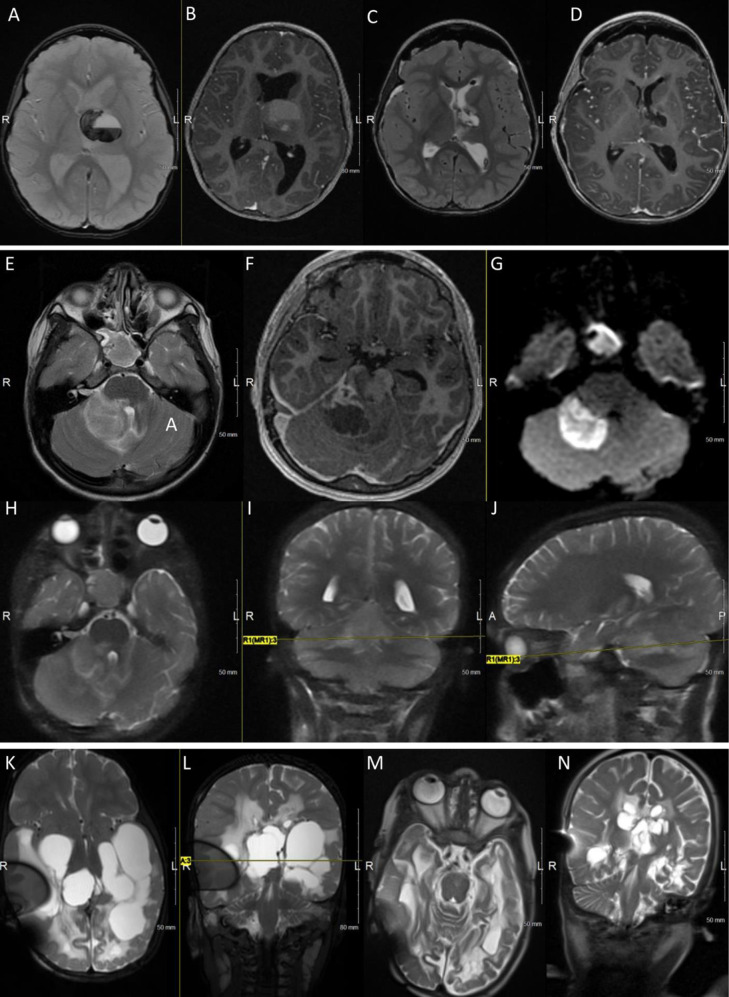
Illustrative cases of non-tumor pathologies.

Between 2018 and 2023, 22 pediatric patients underwent 27 procedures. The patients’ ages ranged from 10-month-old to 19-year-old (mean 9.61 years-old, median 8 years-old). The cohort included 10 females and 12 males, suffering from a variety of lesions: 13 tumors, 5 cavernomas, 1 colloid cyst, 1 cerebellar abscess, 1 traumatic ICH and 1 resection of choroid plexus causing CSF over production. (Table 1, Fig 1-2).

Patient 6, A-D: A. T1 post-contrast sagittal MRI view demonstrating a solid mass in the roof of the third ventricle with obstructive hydrocephalus. B, C. T2-weighted sagittal and coronal MRI views demonstrating the mass and the associated hydrocephalus with aqueductal stenosis. D. Post-resection, T1 post-contrast sagittal MRI view, with a limited residual mass and re-establishment of CSF outflow path. Patient 10, E-L: E. Pre-operative T1 post-contrast axial MRI view demonstrating a large contrast-enhancing lesion, with solid and cystic components, compatible with an atypical meningioma (WHO II). F-H. Axial, sagittal and coronal T2-weighted images showing the fMRI findings (yellow- language areas; green – corticospinal tract; dark blue – inferior longitudinal fasciculus; white – arcuate fasciculus). I-L. T2-weighted and T1 post-contrast coronal, sagittal and axial MRI views obtained 2 years after the MIPS procedure (and shunting), with no evidence of residual tumor and well decompressed ventricles. Patient 12: M-P: M, N. Pre-operative T1 post-contrast axial and coronal MRI views showing an enhancing lesion abutting the right corticospinal tract with local mass effect on the temporal lobe and mesial structures. O, P. T1 post-contrast axial and coronal MRI views with a minimal residual enhancing rim along the corticospinal tract.

Patient 8, A-D: A. Axial MRI gradient echo (GRE) view showing a left thalamic hemorrhagic lesion; B. T1 post-contrast axial MRI view depicting a left thalamic suspected cavernous malformation. C, D. Intra-operative T2-weighted and T1 post-contrast MRI images obtained after the resection of a cavernous malformation. Patient 13, E-J: E-G. T2-weighted, T1 post-contrast and diffusion-weighted axial MRI images showing a new cerebellar abscess in a patient with Streptococcus Intermedius sinusitis. H-J. Post-MIPS axial, coronal and sagittal fast T2-weighted MRI sequence showing no evidence of residual abscess with minimal signal changes along the lateral trans-cerebellar tract. Patient 14, K-N: K, L. T2-weighted axial and coronal MRI images showing post-infectious loculated hydrocephalus. M-N. T2-weighted axial and coronal MRI views obtained over two months after bilateral fenestrations and shunting showing collapsed (communicating) cystic areas with resolved mass effect on the brainstem.

The smaller BrainPath sheath (11 mm in diameter) was used in several cases, including: evacuation of a cerebellar abscess (Patient 13), evacuation of an intracranial hemorrhage (ICH, Patient 17) and hydrocephalus related procedures - resection of cerebrospinal fluid (CSF) over-producing choroid plexus (Patient 15) and fenestrations in post-infectious loculated hydrocephalus (Patient 18, bilateral procedures). Pre-operative 5-ALA was administered in two cases (Patients 9 and 12). Patients 10 and 22 required switching to longer BrainPath sheaths during resection of intraventricular tumors due to collapse of CSF spaces and deepening resection. One patient with post-IVH post-infectious cystic hydrocephalus underwent 4 planned procedures for cysts fenestrations.

The postoperative follow-up averages 701 days (23.36 months). The average length of the procedure was 213 minutes (skin-to-skin) in all the cohort. When calculating for patients not undergoing intra-operative MRIs, the average length of procedure was 144 minutes (2.4 hours).

In 13 cases an intra-operative MRI (iMRI) was obtained; in two cases the MRIs were completed prior to closure, suspecting minimal tumor residual, which was further fully resected (Patients 16, 21). The average length of stay was 10 post-operative days for all patients (per procedure; Patient 8 underwent two procedures during the second hospitalization due to suspected minimal residual cavernoma; Patient 18 underwent two planned bilateral procedures during the same admission). The average length of stay per-procedure for the subgroup of patients with an external ventricular drain (EVD) was 15 post-operative days (median: 13 days, range: 2-44 days), those undergoing a total of 15 procedures, and 6 post-operative days (median: 3 days, range: 1-9 days) for patients without an EVD, undergoing a total of 12 procedures. When removing the severe multi-trauma patient (with an expected prolonged hospitalization), the average stay for patients without an EVD was 3 days.

In 7 of 13 patients operated for the resection of tumors, a gross total resection (53.8%) was achieved. In the remaining 6 patients (46.2%), resections ranging from 40% to 80% were obtained. The extent of resection was limited in two patients with large sellar/parasellar WHO I pilocytic astrocytomas (Patients 11 and 14), in one patient with a third ventricular tanycytic astrocytoma (Patient 6, approximately 80% resection) and in one patient with a right thalamic tumor (Patient 12), in which we stopped the resection approximately 2 mm from the cortico-spinal tract based on intraoperative monitoring activations at 2 mAmp. Patient 14 underwent a subsequent additional resection due to progressive growth the astrocytoma (after completion of chemotherapy and over 18 months from the initial MIPS intervention), obtaining a similar resection of approximately 40-50% of the new tumor volume through the existing MIPS tract (“open approach”). To note, the initial MIPS procedure was much better tolerated by the patient, with a significantly shorter recovery and stay, despite the younger age at the time of the intervention. In two patients with H3K27-altered diffuse midline glioma (Patients 19 and 20) we obtained resections of at least 40% of the overall tumor burden: in the first patient over 50% of the enhancing mass, and, in the latter patient, close to 80% of the enhancing mass. The first patient treated for an H3K27-altered diffuse midline glioma was an urgent procedure for decompression and evacuation of presumed actively hemorrhagic tumor with clinical presentation of herniation; this procedure was performed in the middle of the night, without available monitoring or iMRI support, likely limiting the extent of resection (lifesaving procedure). In the second patient, the infiltrative enhancing tumor was considered to involve midline structures, with T2 hyperintense signal extending from the internal capsule to the medulla and to the cranio-cervical junction. Both patients expired from progressive disease, one 2.3 months after the procedure and the second 5.5 months post-surgery. In one case (Patient 11, middle cranial fossa pilocytic astrocytoma, WHO I), MIPS was superior to the previously performed open approach, resulting in an additional 50% resection of the overall tumor volume, compared to the only 30% achieved through a previous extended orbito-zygomatic approach. The recovery after MIPS was also significantly shorter and better tolerated by the patient, without any additional neurological deficits.

One patient (Patient 9) underwent an awake procedure with intraoperative monitoring for speech and memory for the resection of a peri-forniceal cavernoma. The intraoperative monitoring allowed us to complete a gross total resection (GTR) with no cognitive or memory deficits. 5-ALA was administered preoperatively in a 4-year-old patient (Patient 12), positively illuminating a WHO 1 pilocytic astrocytoma, assisting in the resection of 70-80% of the right thalamic enhancing mass; the resection of the lesion was stopped after identifying cortico-spinal activations at 2 mA stimulation, thus preventing any adverse outcomes.

Patient 8 underwent two re-operations using MIPS for additional resection of a minimally residual cavernous malformation located in the left thalamus. A contralateral right frontal approach was used during all interventions, utilizing the same tract. The iMRI completed at the end of the initial resection did not reveal any residual abnormal tissue, including no evidence of residual cavernoma, however, during the routine follow-up, several months later, we identified a suspected small rebleed that was initially managed conservatively. Approximately 10 months after the first intervention the patient re-presented with headaches and irritability, most likely secondary to additional intracavitary bleeding. A similar approach was used a second time for clot evacuation, as well as for resection of a small solid component consistent with a cavernous malformation. During the same admission, the patient underwent an additional identical procedure due to suspected minimal residual on the post-operative MRI (more conspicuous after the initial evacuation of the hemorrhagic component). The patient was discharged home on POD 3, with no evidence of residual mass or re-bleed during over two years of follow-up.

In our treated population, three patients required a new post-MIPS ventriculoperitoneal shunt (VPS) (Patients 10, 14 and 19), the first due to a trapped left temporal horn, after a gross total resection of an atypical meningioma located in the atrium of the left lateral ventricle (delayed presentation of hydrocephalus), with no evidence of tumor recurrence after 26 months of follow-up. The second patient underwent placement of a VPS approximately 15 months post-op due to progressive hydrocephalus secondary to slow tumor growth during chemotherapy. Patient 19, who initially presented with intra-lesional hemorrhage and clinical herniation, subsequently diagnosed with a diffuse midline glioma H3K27-altered, developed radiological evidence of hydrocephalus with very minimal clinical recovery (palliative care, deceased).

Patient 15 underwent resection of approximately 90% of the left lateral ventricle choroid plexus. Briefly, the patient presented shortly after birth with severe ventriculomegaly in the setting of Coffin-Siris syndrome. An initially performed endoscopic third ventriculostomy (ETV) failed within several weeks, requiring the placement of a ventriculoperitoneal shunt. The patient did well for several months, however, presented with severe ascites, presumed to be secondary to excessive CSF over production (over 900 mL per day, estimated). Considering the large ventricles and diminished parenchymal mantle, we opted to use a tubular retractor for an extensive resection of the choroid plexus of the left lateral ventricle, with no complications, including no evidence of shunt obstruction (contralateral catheter, not externalized the at the time of the MIPS intervention). After over 12 months of follow-up, the patient continued to do well, requiring gradually increasing shunt pressures (proGAV 2.0 Valve^®^, Aesculap, USA) due to over drainage and the initial formation of subdural CSF collections (resolved, valve settings increased from 0 to 13 cmH_2_O).

There were no long-term or permanent new neurological deficits attributed to the minimally invasive surgical procedures. Post-operatively, the return to baseline pre-operative neurological condition was immediate, and, subjectively, the MIPS procedures were better tolerated by the patients.

## Discussion

The use of minimally invasive procedures in neurosurgery, both for cranial and spine pathologies, continues to be broadly adopted. The inherent advantages of tubular retractors, associated with minimal collateral damage to healthy tissue (possibly by dispersing retraction forces) [10–12], and the reported shorter recovery times, are well recognized [13,14]. However, the benefits and best practices of using MIPS in pediatric population are not well characterized. The youngest treated patient in our cohort was 10 months-old, having an excellent outcome and no complications. Eleven procedures were completed in patients 5 years of age and younger, all with excellent outcomes and no post-operative complications, demonstrating the feasibility and safety of the approach in this population.

In our series we achieved a 53.8% GTR rate for tumors (7/13 patients), sub-total resections in 4 patients (30.7%) and partial resections in 2 patients (15.3%). In a large subgroup of patients, the preliminary frozen pathology results played a role in decision making regarding the intended extent of resection, as the low-grade pathologies in the pediatric population (such as pilocytic infiltrative tumors) have a good outcome even in cases when a GTR cannot be safely obtained, especially considering the possible benefits from novel targeted chemotherapy. The use of a tubular retractor can be highly beneficial when a partial resection is targeted to determine pathology (obtaining large and sufficient volumes of tissue for molecular analysis), resolving obstructive hydrocephalus or decreasing intracranial pressure.

The overall GTR rate for all intracranial lesions (including tumors, cavernomas and a colloid cyst) was 66.67% (12/18 patients). In a sub-analysis, the GTR rate for exophytic intraventricular lesions (7 tumors, one cavernoma and one colloid cyst) was 88.8%. When considering cavernomas, the GTR rate was 100%. One colloid cyst was also fully resected using MIPS, after a previously failed endoscopic resection.

*Eichberg et al.* [4], reported a GTR rate of 71.7% in a series of 113 transtubular resections in the adult population, with a permanent complication rate of 4.4%. The series included different pathologies approached and treated in the adult cohort: metastasis (23.9%), glioblastoma multiforme (23.0%) and cavernous hemangiomas (22.1%), with a mean lesion depth of 4.4 cm. In a literature review by *Shapiro et al.* [14], the GTR rate for tumors using the Vycor™ retractor system (Vycor Medical Inc., Boca Raton, Florida, USA), alone or in combination with other retractors, was 63%, with 3 short-term post-operative complications related to the retractor. Considering the differences between the nature and prevalence of different types of lesions encountered in the adult population compared to the pediatric ones (such as metastases in adults versus complex and large pediatric sellar/suprasellar low grade tumors), the average depth of those tumors from the cortical surface, as well as the different oncologic goals of the procedures, one can argue that our reported outcomes are at least similar if not superior to the adult experience. We did not encounter any measurable complications related to the retractors or the surgical procedure.

For pediatric patients presenting with unique aspects, such as a more prominent friability of younger brain tissue and possible catastrophic long-term sequela from surgical-related infections, the significance of reduced surgical time is a key MIPS attribute: 55% of the procedures in our series were completed in less the 3 hours, with an average time of 144 minutes (2.4 hours) for procedures without an intra-operative MRI.

We performed complex resections in an acute setting, shortening the surgical time, while promptly debulking large lesions, controlling hemorrhage and effectively decreasing ICPs in pediatric patients presenting with various levels of herniation. Despite a very poor outcome in one patient with acute intra-tumoral hemorrhage and clinical herniation, the tubular retractor allowed for immediate control of the hemorrhage and hematoma evacuation, followed by significant tumor debulking of over 50% of the enhancing mass. Despite the lack of neuromonitoring and performing the procedure in the middle of the night, the post-operative MRI demonstrated preserved presumed functional areas, likely due to a fixed working corridor and orientation, based on a navigated trajectory. A second patient, presenting with a similar lesion with radiological evidence of subfalcine herniation and sub-acute progressive hemiparesis, underwent a successful resection of 70-80% of a thalamic enhancing mass, benefiting from improving hemiparesis in the immediate post-operative course. Only one out of the 22 patients (Patient 8) had an unexpected outcome in terms of residual cavernoma (inconspicuous on the post-operative MRI), requiring return to the operating room, using an identical approach and instrumentation. In all other patients the intervention yielded the expected and even possibly superior outcomes. When accounting for non-trauma patients without an EVD, the average length of stay was 3 post-operative days, which is relatively short for a pediatric population.

**Fig 3:**
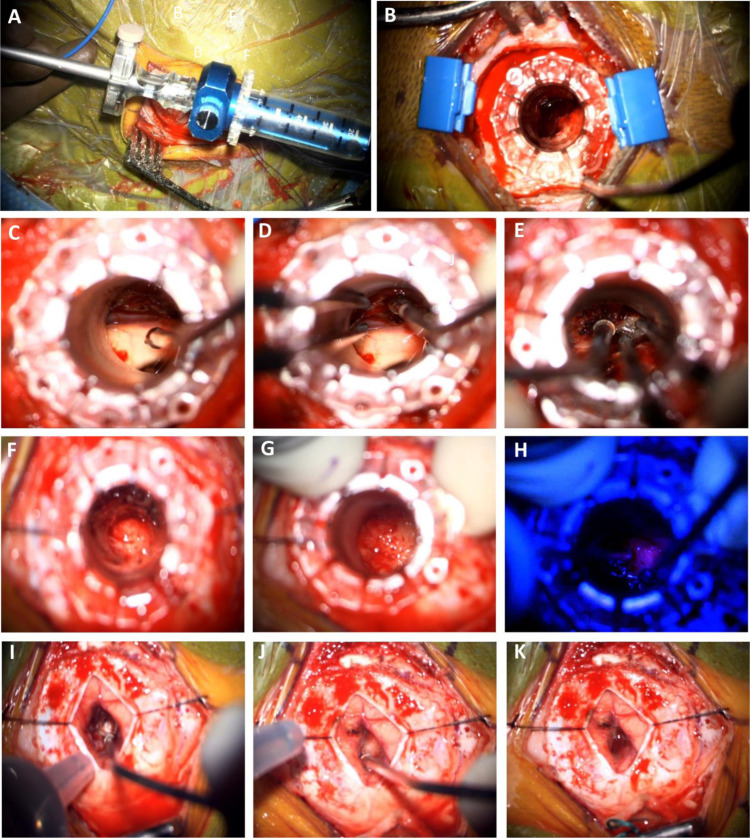
Intraoperative images.

The use of tubular retractors in the pediatric population, even though similar to the implementation in adult patients, carries several unique aspects. The first one is associated with the limitations on rigid head fixation in young children. For patients younger than 3-4 years-old, we performed our earliest cases using the Stealth electromagnetic navigation stylette secured with bone wax to the obturator. This could have been associated with sub-optimal accuracy. As the company introduced the dedicated adaptor for electromagnetic pointers (BrainPath Navigation Probe Adapter^®^, Figure 3), our workflow and accuracy has improved. An additional significant difference that can impact the safety and success of the procedure is the size of the craniotomy in relation to the thickness of the bone. We found that in young patients with thin bone, a smaller craniotomy was sufficient to allow satisfactory dynamic angulation of the tubular retractor. As the thickness of the bone increases with age, either a larger sized craniotomy must be completed, or a longer tube has to be utilized (with the inherent disadvantages), thus, to avoid the interference between the rim of the sheath and the cranium. Lastly, special care must be given to the more friable pediatric brain, occasionally with a thinner cortical rim due to associated conditions such as hydrocephalus. Considering the additional lower overall intracranial pressures, there is a possibly higher risk of inward pulling of a larger cortical surface if the pia has not been meticulously and sufficiently fenestrated. This can be associated with persistent post-operative extra-axial hygromas. Therefore, the opening in the pia in the younger patients should be slightly more extensive, considering the use of the smaller diameter BrainPath when technically safe for the specific pathology.

A. BrainPath sheath and obturator with BrainPath Navigation Probe Adapter^®^; B. Low magnification view of a 13.5 mm tubular retractor in relation to the craniotomy size. C-E. Resection of a left thalamic cavernoma (Patient 8). F-K. Resection of a right basal ganglia pilocytic astrocytoma (Patient 12): F. Early intra-operative view of the lesion. G, H. Positive 5-ALA illumination (H), demonstrating the lesion in the right lateral quadrant (G). I-K. The size of the cortical corridor and its immediate collapse following the removal of the tubular retractor.

### Technical aspects of the MIPS procedures in the pediatric population

The number and variety of pediatric cases completed using this approach, as well as the analysis of outcomes yielded multiple observations and technical insights. One of the benefits of using a tubular system consists of possibly reducing the collateral damage to the healthy brain tissue along the tract [5]. This can be more significant with deeper lesions and when the approach is in close proximity to functional areas and tracts [15]. From a practical perspective, working through a fixed and continuously protected surgical corridor minimized the risks of “deviation” from the determined safe trajectory and injury to or excessive manipulation of uninvolved structures.

Previous reports [16], emphasized the advantage of the small craniotomies required for the completion of this procedure. While appropriate for lesions such as ICH, in our experience, when approaching large tumors, the ability to partially manipulate and changing the angular orientation of the sheath was critical, thus allowing for larger resections. Therefore, we recommend the completion of a wider diameter craniotomy, at least 1.5-2 times larger than the outer diameter of the sheath.

Considering the diversity of the treated pathologies, we resected both soft tumors, using mostly the standard suction and bipolar technique, but also intraventricular meningiomas, with significant calcifications. In the latter case, as well as in others, such as a third ventricular tumor and large astrocytomas, the use of the illuminated Myriad^®^ debrider and/or the ultrasonic aspirator (using a micro-tip to allow better visualization), were extremely helpful [17].

With experience, we found the longer sheaths to be superior, as they allowed maintenance of the corridor even as the brain collapses secondary to CSF drainage and tumor debulking. This requires experience and surgeon’s comfort using longer instruments throughout the entire procedure, and, even though we were able to safely replace the sheath during several interventions, this step can and should be avoided. In our institution, we assembled dedicated surgical trays, that include long and thin instruments (tumor forceps, micro scissors, micro dissectors, etc.), that can be used through the longest sheaths, while minimizing the interference to the line of sight and the amount of deep illumination from the visualization system in use. Availability of long suctions and bipolars is critical.

Intraoperative navigation is an intrinsic part contributing to the success of those interventions. A more unique aspect of neuronavigation in the very young pediatric population consists in limitations using a rigid head fixation system, therefore requiring the use of electromagnetic navigation. Earlier procedures completed in the younger patients required adaptation of the Stealth™ electromagnetic (EM) stylet to navigate the BrainPath complex. The more recent availability of the dedicated adaptor for this system (BrainPath Navigation Probe Adapter^®^) improved the workflow and the overall accuracy. We also found that the longer tracer pointer designed for rigid frame-based navigation of shunt catheters (versus the shorter tracker) was more beneficial, and its use should be considered during the registration step.

Preoperative trajectory planning is crucial [18], in parallel to defining accurate and appropriate goals for the intended procedure. Pre-operative identification of safe corridors (based on DTI and basic functional anatomy), as well as the best angle of approach to the lesion, given the limited ability to manipulate the sheath, can improve and optimize the line-of-sight coverage of the entire lesion [15]. Expected good dissection planes versus areas of blood supply or tumor origin should be taken into consideration when planning the trajectory of the tubular retractor.

Intra-operative cortical/subcortical mapping was effectively performed when indicated. In our experience, when subcortical mapping was implemented, the extent of resection of a thalamic pilocytic astrocytoma abutting and displacing the corticospinal tract was maximized, with similar expected efficacy compared to standard open approaches.

Awake procedures are feasible with MIPS and, possibly, even better tolerated, considering the smaller incision, craniotomy, dural opening and shorter length of procedure. However, extensive mapping is not as feasible with this approach, unless performed cortically and only for determining safe entry areas.

In addition, our experience has shown that the use of adjuncts such as 5-ALA was feasible, with proven benefit when identifying positive immunofluorescence, contributing to a safe maximal resection (Patient 12).

For Patient 6, we used a flexible endoscope inserted through the tubular retractor to evaluate the intraventricular anatomy, blood supply to the tumor, relationship to major vascular structures and residual mass, providing valuable information used for critical decision making. We recognize that some of the reported procedures could have been completed endoscopically. The authors believe that, given the complexity of several of the treated lesions, a purely endoscopic approach would have yielded a less optimal outcome even in very experienced hands. Moreover, comparing the diameter a typical peel-away tunneler (for example, 19 Fr), a tubular retractor that is only 11 mm in diameter allows the use of “standard” microsurgical instruments and the safe completion of the procedure by a single neurosurgeon, without the need of an experienced assistant.

Lastly, in addition to the previously discussed critical aspects of meticulous presurgical planning [15], the use of dedicated tools and instruments, while still implementing classical neurosurgical adjuncts (monitoring, 5-ALA, etc.), the learning curve of a new surgical approach cannot be underestimated. We recommend starting with relatively simple procedures and pathologies, such as ICH and metastasis, or superficial lesions where a transition to an open approach can be easily obtained. Evaluation of the expected intraoperative complications should be performed, constantly assessing the possibility of critical hemorrhage and the ability to control it through a small and fixed working corridor. As previously mentioned, pre-operative planning in relation to the expected origin of blood supply to the lesion is critical. In this regard, we favor the completion of preoperative neurovascular imaging if a vascular lesion is included in the differential diagnosis, possibly considering alternative surgical approaches. The availability of extra-long and high quality bipolars, as well as constant evaluation of the actual and possible (realistic) line-of-sight cannot be over-emphasized. We recommend working in defined compartments with the widest possible field of view. The use of micro brain patties as well as various hemostants have been highly efficient in our experience. Use of endoscopy for better evaluation of the lesional architecture and possibly controlling remote focal hemorrhagic areas should be considered (and readily available). The sheath length should be carefully considered: a too long tube will significantly increase the instruments manipulation difficulty, and a too short tube will not maintain the surgical corridor, allowing the surrounding tissue to collapse into the surgical field. A team-based approach, shared expertise, as well as slowly and carefully increasing the complexity of the performed procedures, played a significant role in our results. Risk assessment and management, as well as realistic surgical goals, should be constantly taken into consideration, even when limiting the extent of resection.

## Limitations

Our reported experience has several limitations. First, we were unable to complete an accurate comparison to similarly heterogeneous pathologies and patients treated using the “standard of care” approaches. Thus, comparing complication rates, length of stay, overall recovery time, rate of re-operation or the need for additional treatments and interventions (such as shunting, chemotherapy, etc.) to traditional procedures was not feasible. Although our cohort is the largest report consisting of pediatric patients who had undergone minimally invasive interventions using this tubular retractor system, comparison of outcomes based on a sub-classification of different pathologies was not attainable due to the small sample size. Additionally, quantifying the extent of injury to healthy tissue and comparing it to the traditional approaches is extremely difficult. Even though we did not encounter complications related to the tubular retractor and surgical technique, temporary or permanent deficits are more difficult to assess in pediatric patients. The length of stay was also significantly affected by the complexity of some of the lesions and the different pediatric management routines, such as the presence of an EVD with a slow weaning process, or interdisciplinary management of multi-trauma patients.

## Conclusion

Our initial results and experience in the treatment of a variety of pediatric pathologies using minimally invasive techniques are very promising. As with every aspect of neurosurgical practice, appropriate patient selection and individualized approaches, meticulous presurgical planning, intraoperative flexibility, in lieu of surgeon’s experience, foster the best outcomes and minimize complications. Developing technical familiarity and surgical experience, in addition to the use of appropriate instruments and visualization systems and, as well as the implementation of adjunctive tools, are critical for the success of minimally invasive interventions and contribute to superior outcomes. Additional data on safety and long-term outcomes is required to better define the role of MIPS for pediatric intracranial pathologies.
